# CRPS of the upper or lower extremity: surgical treatment outcomes

**DOI:** 10.1186/1749-7221-4-1

**Published:** 2009-02-20

**Authors:** A Lee Dellon, Eugenia Andonian, Gedge D Rosson

**Affiliations:** 1 Plastic Surgery and Neurosurgery, Johns Hopkins University, Baltimore, Maryland, Suite 370, 3333 N. Calvert St. Baltimore, Maryland, 21218, USA; 2 Dellon Institute for Peripheral Nerve Surgery: Baltimore, Maryland, Suite 370, 3333 N. Calvert St. Baltimore, Maryland, 21218, USA; 3 Division of Plastic Surgery, Johns Hopkins University, Baltimore, Maryland, Suite 370, 3333 N. Calvert St. Baltimore, Maryland, 21218, USA

## Abstract

The hypothesis is explored that CRPS I (the "new" RSD) persists due to undiagnosed injured joint afferents, and/or cutaneous neuromas, and/or nerve compressions, and is, therefore, a misdiagnosed form of CRPS II (the "new" causalgia). An IRB-approved, retrospective chart review on a series of 100 consecutive patients with "RSD" identified 40 upper and 30 lower extremity patients for surgery based upon their history, physical examination, neurosensory testing, and nerve blocks. Based upon decreased pain medication usage and recovery of function, outcome in the upper extremity, at a mean of 27.9 months follow-up (range of 9 to 81 months), gave results that were excellent in 40% (16 of 40 patients), good in 40% (16 of 40 patients) and failure 20% (8 of 40 patients). In the lower extremity, at a mean of 23.0 months follow-up (range of 9 to 69 months) the results were excellent in 47% (14 of 30 patients), good in 33% (10 of 30 patients) and failure 20% (6 of 30 patients). It is concluded that most patients referred with a diagnosis of CRPS I have continuing pain input from injured joint or cutaneous afferents, and/or nerve compressions, and, therefore, similar to a patient with CRPS II, they can be treated successfully with an appropriate peripheral nerve surgical strategy.

## Background

For the patient given the traditional diagnosis of "Reflex Sympathetic Dystrophy" (RSD) who fails to recover from sympathetic blocks, anti-inflammatory medication and physical therapy, current treatment options largely consign the patient to a Pain Management center for life.[1,2] Changing the diagnosis to the more "appropriate", current term, "Complex Regional Pain Syndrome I" (CRPS I),[3] does not change this treatment plan.[1,2] Other than sympathectomy or an implanted spinal cord or peripheral nerve stimulator, surgery is rarely recommended.[1,2,4-6] For the lower extremity, "RSD of the knee", [7-11] and CRPS I of the foot[12] have been characterized, but, again, without a suggestion that surgical intervention on the peripheral nerve itself might be appropriate.[13] In contrast, the traditional "causalgia", now termed "CRPS II", is by definition pain related to a peripheral nerve injury, and, if the appropriate source of that pain generator were identified, then a peripheral nerve surgical strategy would be considered appropriate.

Peripheral nerve surgery has been considered an option in the upper extremity for the diagnosis of co-existing carpal tunnel syndrome once the patient has passed the acute phase of pain and associated swelling.[2,14-16] Recently, a single case report has described relief from "CRPS I" for a patient who required, in addition to the carpal tunnel decompression, a nerve graft reconstruction of an injured radial sensory nerve after a distal radius fracture.[17] A single case of re-excision for a multiply-recurrent "Morton's neuroma" and "RSD" has been reported.[18] These few patients raise the concept that the pain generating source(s) in patients with "CRPS I" might be due to the presence of undiagnosed peripheral nerve injuries or compressions, which would change the diagnosis from CRPS I to CRPS II, and, therefore, change the prognosis and therapeutic plan. If recurrent carpal tunnel syndrome has been approached surgically for the patient with "CRPS I", would it not be possible for appropriate surgical intervention to be considered if a source of pain from wrist, [19-21] knee,[22] or ankle joint afferents[23] could be identified in the patient with "CRPS I"?

With the hypothesis that chronic pain input to the dorsal spinal columns can be the source of CRPS II, misdiagnosed as CRPS I, and with the hypothesis that injured joint and/or cutaneous afferents as well as chronic nerve compression can be the source of these painful dorsal column inputs, an approach was taken to re-evaluate patients with CRPS I of the upper or lower extremity. The results of this approach are now reported.

## Methods

An IRB-approved, retrospective chart review was carried out on a series of 100 consecutive patients with the diagnosis of "RSD" from a computer database from 01-01-2000 through 04-30-2006. Diagnosis was made based upon the International Association for Study of Pain criteria:[3]*Absolute*: pain extending outside the area of trauma, impaired extremity function, and either cold or warm perceptions or temperature changes in the affected extremity; *Relative: *swelling, increased hair or nail growth, hyperalgesia, allodynia, abnormal skin coloring.

Inclusion into the surgery group required 1) failure to have the chronic pain resolve despite more than 6 months of therapy, edema control, anti-inflammatory medication, and opiates under the control of a pain management physician, 2) documentation of a pain input source by nerve blocks of joint and/or cutaneous afferents, with a ≥ 5 point reduction in pre-block pain on a Visual Analog Scale (score of 0–10, with 10 being the worst pain), and 3) documentation of chronic nerve compression by abnormal neurosensory testing and the presence of a positive Tinel sign at the known site of anatomic narrowing (due to patient pain levels, electrodiagnostic testing was not an inclusion criteria). Based upon these inclusion criteria, seventy patients from the 100 were selected for surgery, 40 patients had upper extremity and 30 patients had lower extremity CRPS.

The nerve block was done using a 50:50 mixture of 1% xylocaine and 0.5% bupivicaine, each without epinephrine. Sterile technique was used. For each nerve block, 5 cc of this mixture was infiltrated into the region of the known joint afferent or suspected cutaneous neuroma. The joint itself was not infiltrated. The nerve itself was not knowingly injected. Sites of suspected nerve compressions were not injected to avoid increasing the pressure on the compressed nerve. Failure of the nerve block to relieve pain could imply that the block was not done properly, and therefore "failure" meant that while loss of sensation did occur in the distribution of the sensory nerve that was blocked, there was insufficient pain relief to deem that block successful. When this occurred, it was still possible that another nerve contributed to the pain mechanism and that the two sensory territories overlapped, e.g., the radial sensory nerve and lateral antebrachial cutaneous nerve, or the sural and superficial peroneal nerve territories. This anatomic possibility was investigated by a new block of the suspected adjacent nerve. If the pain remained after nerve block of all possible sensory nerve territories, then failure to obtain pain relief was interpreted to mean that there was a fixed central mechanism (dorsal column, thalamus) for the pain and that peripheral nerve surgery would not be of benefit.

Patient demographics are given in Table 1.

**Table 1:** Demographics

	Age mean, range (yrs)	Interval Post-Injury mean, range (mo.)	Follow-Up mean, range (mo.)
**Extremity**			

**Upper**	49.5 (21–82)	45.4 (6–192)	27.9 (9–81)

**Lower**	35.3 (24–73)	56.5 (4–249)	23.0 (9–69)

The distribution of the mechanisms of injury in each group is given in Table 2.

**Table 2:** Injury mechanism

	Work Injury	MVA	Iatrogenic	Personal Injury
**Extremity**				

**Upper**	54%	5%	27%	14%

**Lower**	36%	6%	29%	29%

In patients with either 1) a history of repeated swelling or acute exacerbations of pain after extremity use, 2) evidence of persistent sympathetic over-activity, or 3) pre-emptive anesthesia was requested by the patient's Pain Management doctor, the surgery was done using indwelling brachial plexus[24] or epidural catheter plus general anesthesia.[25]

The distribution of surgical procedures done is given in Table 3. Most patients required more than one nerve to be treated surgically. The surgical techniques have been described previously for joint denervation of the wrist,[19,26] elbow,[27] knee, [28-30] and ankle.[31,32] The surgical technique for resection of cutaneous neuroma and muscle implant has been described previously for the radial sensory and lateral antebrachial nerves,[33] for the medial antebrachial nerve,[34,35] for the posterior cutaneous nerve of the forearm,[36] superficial and deep peroneal nerves,[37] for the saphenous nerve,[38] and for the calcaneal nerves.[39]

**Table 3:** Number of operations of each type required*

	Neurolysis	Joint Denervation	Neuroma Resection
**Extremity**			

Upper**	25	5	19

Lower***	27	9	15

* total numbers are greater than number of patients: most patients required more than one nerve resected and/or denervation and/or neurolysis to achieve relief

** neurolysis included the brachial plexus (2), carpal (5) and cubital tunnel (9) and radial sensory (9) nerves, while denervated joints included the elbow (1) and wrist(4), and neuromas were resected from the radial sensory (5), lateral antebrachial (7), posterior cutaneous nerve of the forearm (3), medial brachial (1), medial antebrachial nerves (2), and a digital nerve that was reconstructed with a conduit.

*** neurolysis included the common peroneal (10), superficial (5) and deep peroneal (1) nerves, interdigital nerve (1) and release of the four medial ankle tunnels for the tibial nerve (10), while denervated joints included the knee (5), and ankle (4), and neuromas were resected from the superficial peroneal nerve (4), saphenous (6), sural (2) and medial calcaneal nerves (3).

### Statistical methods

Outcomes included measurement of pain, level of drug use, improved function as determined by activities of daily living and return to work. An excellent result required the pain level to be from 0 to 2, a cessation of opiate use, the ability to use the upper or lower extremity for normal activities of daily living, and the return to work (if previously employed) or school. A good result required the pain level to be from 3 to 4, an occasional use of a non-opiate, a slight restriction of activity of daily living, and the return to work (if previously employed) or school. All impairments greater than the above criteria were considered a failure. Descriptive statistics were used for the demographics.

## Results

In order to evaluate whether there was a difference in outcomes between patients with a relatively short length of follow-up and those with a longer length of follow-up, the patient population was grouped into those whose follow up was ≥ 9 but ≤ 24 months, and those whose follow-up was > 24 months.

### Entire series

In the upper extremity the results were excellent in 40% (16 of 40 patients), good in 40% (16 of 40 patients) and failure 20% (8 of 40 patients).

In the lower extremity the results were excellent in 47% (14 of 30 patients), good in 33% (10 of 30 patients) and failure 20% (6 of 30 patients).

### Follow-up > 9 and < 24 months

In the upper extremity the results were excellent in 35% (9 of 26 patients), good in 38% (10 of 26 patients) and a failure in 27% (7 of 26 patients).

In the lower extremity the results were excellent in 41% (7 of 17 patients), good in 35% (6 of 17 patients) and a failure in 24% (4 of 17 patients).

### Follow-up > 24 months

In the upper extremity the results were excellent in 50% (7 of 14 patients), good in 43% (6 of 14 patients) and a failure in 7% (1 of 14 patients).

In the lower extremity the results were excellent in 55% (7 of 13 patients), good in 30% (4 of 13 patients) and a failure in 15% (2 of 13 patients).

There was no statistically significant difference between results in the upper versus the lower extremity or between any of the three time-frame groupings.

## Discussion

This study demonstrated that more than 80% patients with a diagnosis of "CRPS I" of the upper or the lower extremity have one or more injured and/or compressed peripheral nerve(s) as the source of their continuing dorsal column painful neural input. This means that perhaps as many as 80% of patients with the current diagnosis of "CRPS I" should actually have a diagnosis of "CRPS II", and provides optimism that a peripheral nerve source for their pain can be identified and successfully treated using an appropriate peripheral nerve strategy.

This study demonstrates that the accepted principles of upper extremity peripheral nerve surgery apply to the lower extremity. If nerve decompression of the median nerve in the carpal tunnel can be accomplished safely for the patient with CRPS I,[14] then nerve decompression should be possible and safe in the lower extremity for the patient with tarsal tunnel syndrome. If the principles described 20 years ago for partial wrist denervation continue to be applied successfully today for wrist pain,[40] then it should be safe to apply this approach for patients with ankle injury whose ankle pain can be relieved by a block of the deep peroneal nerve. If the principles described in the past to treat painful cutaneous neuromas in the upper extremity can be applied successfully in the lower extremity, then patients with CRPS I after ankle injury, who have persistent superficial peroneal and sural neuromas related to lateral ankle stabilization or fracture fixation, should receive a nerve block of these nerves, and be considered potential surgical candidates.

Painful dorsal column input can arise from injured joint afferents caused by wrist or ankle sprain/dislocation, arthroscopy, or total joint replacement. This source of pain can be determined by nerve block of the appropriate nerve(s). If the nerve block reduces the pain level by ≥ 5 on the Visual Analog Scale, partial joint denervation should be considered. For the wrist, partial denervation can be achieved by resecting the anterior and the posterior interosseous nerves.[19,26] For the dorsolateral ankle, either the deep peroneal nerve above the ankle and/or the sural must be resected[31,32] (approximately 25% of patients have dual innervation of the sinus tarsi).[23] For the knee joint, both the medial and the lateral retinacular nerves must be resected. [29-31]

Painful dorsal column input can arise from neuromas of cutaneous nerves, which can also be determined by nerve block of the appropriate nerve, taking care not to place the anesthetic where two nerves will be blocked simultaneously. If the nerve block reduces the pain level by ≥ 5 on the Visual Analog Scale, resection of the nerve(s) should be considered with implantation of the proximal end into a muscle with minimal excursion.

Patients with "RSD" of the Knee [3-5] should be considered to have a combination of injured joint afferents (the medial and lateral retinacular nerves) and injury to the infrapatellar branch of the saphenous nerve. If these nerves can be demonstrated to be the source of the pain by nerve blocks and post-block increase knee function, then "RSD of the Knee", then "CRPS I" of the knee is really a "CRPS II" of the knee and can be helped by partial knee denervation and resection of the infrapatellar branch of the saphenous nerve. [28-30]

The group of patients with a poor result from this surgical approach, and those in the initial cohort identified by our computer search who were not selected for this peripheral nerve surgical approach, may be concluded to have CRPS I. It is possible that those in our failure group have a fixed central nervous system site for their pain generator, making them unresponsive to peripheral nerve surgery, even if they obtained some relief from a peripheral nerve block.

While there is no doubt that patients with CRPS have impairment in their daily life, documenting this degree of disability is difficult. One approach is to use validated "instruments", or questionnaires to do this. A limitation of the present study is that it did not use a validated outcome instrument to measure the patients before and after their surgical treatment. The outcomes used to assess the results of the surgical interventions in this small group of upper and lower extremity patients with CRPS II do suggest that the appropriate surgical intervention can impact each patient's quality of life, and do this with a low risk of increasing their impairment. The results of the present study suggest that it would be appropriate for a prospective study to utilize both a generalized health-related quality of life instrument as well as one uniquely related to upper extremity function and lower extremity function. The first paper to study quality of life issues in patients with CRPS compared the Nottingham Health Profile, the European Quality of Life 5D, and the Sickness Impact Profile 68 to 100 patients with RSD, 33 in the upper and 21 in the lower extremity.[41] Regardless of whether the patient had CRPS in the upper or the lower extremity, the two groups were significantly more in pain, had significantly more sleep problems, had significantly less energy, had significantly less ability to work, had significantly more problems with sexual relationships and with their social lives with the Nottingham Health Profile than did the normal population. As might be expected, while both the upper and the lower extremity CRPS patients had significantly reduced mobility than the normal population, the lower extremity patients had significantly more reduction in mobility than did the upper extremity patients. The European Quality of Life 5D was able to demonstrate that patients with CRPS in their upper extremities had significantly less ability to care for themselves than did lower extremity patients.

## Conclusion

It is concluded that most patients referred with a diagnosis of CRPS I have continuing pain input from injured joint and/or cutaneous afferents, and/or chronic nerve compression(s), and therefore, similar to a patient with CRPS II, they can be treated successfully with an appropriate peripheral nerve surgical strategy. Using nerve blocks to identify patients whose pain is from a peripheral nerve source is critical to decision making with regard to which nerve(s) should be resected.

## Competing interests

ALD has a proprietary interest in the Pressure-Specified Sensory Device™ and owns Sensory Management Services, LLC.

## Authors' contributions

ALD originated the concept of the study, and the surgical procedures, wrote the first and final drafts of the manuscript. EA did the chart data review and analysis, and edited the Manuscript. GDR participated in the surgery, helped conceive the research design and IRB approval, reviewed the data and the manuscript.
